# Sweet Fennel Essential Oil Repels *Drosophila suzukii* via an *Orco*-Mediated Pathway

**DOI:** 10.3390/insects17090957

**Published:** 2026-09-14

**Authors:** Wangyue Zhang, Litong Wei, Jiali Qian, Fengyi Wen, Junnan Yao, Ke Zheng, Yu Zhang, Huiming Wu, Chunxia Tian, Mengli Chen

**Affiliations:** 1 Zhejiang Key Laboratory of Biology and Ecological Regulation of Crop Pathogens and Insects, College of Advanced Agricultural Sciences, Zhejiang A&F University, Hangzhou 311300, China; zwy050322@126.com (W.Z.); 13862715195@163.com (L.W.); 15500926573@163.com (F.W.); 15167023727@163.com (J.Y.); 18258759114@163.com (K.Z.); wuhm@zafu.edu.cn (H.W.); 2 Key Laboratory of Biology of Crop Pathogens and Insects of Zhejiang Province, Ministry of Agriculture Key Laboratory of Molecular Biology of Crop Pathogens and Insects, Institute of Pesticide and Environmental Toxicology, Zhejiang University, Hangzhou 310058, China; jialiqian@zju.edu.cn; 3 Institute of Agro-Product Safety and Nutrition, Zhejiang Academy of Agricultural Sciences, Hangzhou 310021, China; zhyu7711@163.com; 4 Zhejiang Provincial Center for Disease Control and Prevention, Hangzhou 310051, China

**Keywords:** *Drosophila suzukii*, sweet fennel oil, repellency, *Orco*, CRISPR–Cas9

## Abstract

Essential oils are promising alternatives to conventional chemical pesticides due to their toxic, repellent or oviposition-deterrent effects against many kinds of pests. Sweet fennel essential oil is widely recognized for its pharmacological, antimicrobial, and acaricidal properties. Moreover, several studies have demonstrated its insecticidal and repellent activities against insects. In this study, we found that sweet fennel essential oil exhibited strong dose-dependent repellent activity in the dual-choice still-air assay and oviposition behavioral assays against *Drosophila suzukii* adults. Next, we confirmed that trans-anethole was the most abundant constituent in sweet fennel essential oil using GC-MS. In addition, the results of the behavioral assays indicated that trans-anethole displayed comparable repellency against *D. suzukii*. Furthermore, an *Orco* knockout homozygous strain (*Orco*^−/−^) was generated using CRISPR–Cas9 genome editing, and the EAG responses of *Orco*^−/−^ flies to sweet fennel oil and trans-anethole were completely abolished. The repellency elicited by sweet fennel oil and trans-anethole were also dramatically reduced. Taken together, these laboratory results not only reveal that sweet fennel oil holds potential in pest management but also demonstrate that its repellency is mediated by the *Orco* pathway.

## Simple Summary

*Drosophila suzukii* (Diptera: Drosophilidae), a major invasive pest, damages berries and other soft-skinned fruits and causes heavy economic losses. Essential oils, extracted from numerous plant species, exhibit excellent insecticidal and repellent activities against many kinds of pests, which renders them ideal alternatives to chemical pesticides. Here, we found that sweet fennel essential oil exhibited significant repellency against *D. suzukii* in a dose-dependent manner, as well as bioactive constituent trans-anethole in the laboratory. Additionally, to explore whether the repellency was mediated by the *Orco* pathway, we generated the *Orco* knockout homozygous strain (*Orco*^−/−^) using CRISPR–Cas9 genome editing. The results indicated that the EAG responses and repellency were significantly reduced in *Orco*^−/−^ flies, elicited by both sweet fennel essential oil and trans-anethole. Our study demonstrates that sweet fennel essential oil elicits strong *Orco*-mediated repellency against *D. suzukii* adults.

## 1. Introduction

*Drosophila suzukii*, a globally devastating invasive insect pest, causes serious damage to a variety of fruit crops, such as grapes, cherries, berries, and many other soft-skinned fruits. The females possess a specialized serrated ovipositor that can directly pierce the peel of fruits for oviposition. Larvae feed and grow inside the fruits, which may concurrently induce fungal infection, eventually leading to fruit rot and abscission. Consequently, this process causes substantial economic losses for the soft fruit industry, particularly for strawberries and blueberries [1,2]. At present, chemical control is the dominant strategy for field management of *D. suzukii*, and insecticides including cyantraniliprole and deltamethrin are frequently applied in fruit cultivation. However, long-term and sole application of chemical pesticides has resulted in the development of resistance in field populations to various insecticides [3]. Meanwhile, conventional pesticide spraying tends to cause excessive pesticide residue on fruits, failing to meet the safety standards for fresh consumption and export [4]. Furthermore, broad-spectrum insecticides induce high mortality in natural enemies such as parasitic wasps, disrupt the intricate ecological balance of orchard habitats, and diminish the inherent natural regulatory potential among coexisting organisms [5]. The limited availability of chemical pesticides for organic farming, coupled with high costs and short persistence, further exacerbates the difficulties of pest control [6].

As environmentally friendly biogenic pesticides, plant essential oils possess diverse biological activities against a wide array of agricultural, forestry and public health pests. Essential oils exert toxic, repellent or oviposition deterrent effects against many kinds of pests, thus rendering them ideal alternatives to conventional chemical pesticides [7,8,9,10]. For example, Piper essential oil rapidly kills *D. suzukii* adults and suppresses oviposition while exhibiting low toxicity to parasitic natural enemies [8]. In addition, essential oils derived from thyme and lemongrass, as well as their major bioactive components such as carvacrol and citral, effectively repel adult flies and reduce fruit infestation [9]. Previous studies have reported that diverse plant essential oils exhibit both larvicidal activity and adult repellent activity against *Aedes aegypti* [7,10]. Similarly, essential oils from Spanish oregano, laurel or basil significantly inhibit oviposition in lepidopteran pests [11]. These multi-target effects further demonstrate the broad-spectrum management potential of plant essential oils. Meanwhile, the multi-constituent synergistic action of essential oils on insect neural and behavioral targets poses a low risk of resistance, and their eco-friendliness and low non-target toxicity effectively overcome the drawbacks of conventional chemical control [7,8,9,10,11].

Fennel essential oil is widely used in green pest control, food preservation, pharmaceuticals and daily chemicals. It exhibits prominent contact toxicity and repellent effects against sanitary pests such as houseflies [12]. Also, it possesses highly efficient fumigant and contact activity against stored-product pests such as *Tribolium castaneum* and *Sitophilus granarius*, thus serving as an eco-friendly alternative to synthetic insecticides for grain storage protection and pest management in livestock and poultry farming [13]. In the food industry, fennel essential oil is applied as a natural antioxidant or antimicrobial preservative, and its characteristic aroma is also broadly exploited for flavoring [14]. Because fennel essential oil is already permitted for use in food and cosmetic industries, registering it for pest control could be more straightforward than other chemicals and blends, which is a major advantage of using essential oil for pest management. The composition of fennel essential oil differs significantly between its two major chemotypes, sweet fennel and bitter fennel. Sweet fennel contains trans-anethole and fenchone, which imparts a sweet, fresh fragrance suitable for food, daily chemicals and medicinal applications. In contrast, bitter fennel is rich in fenchone and methyl chavicol, with a pungent, bitter odor [12,15]. In addition, plant part, geographical origin, genotype and extraction process all significantly affect the composition of essential oil, and such compositional variation directly determines the strength of its biological activity and its application scenarios [13,14,15,16]. Given that attraction or avoidance behaviors in *D. suzukii* are largely governed by olfactory perception, fennel essential oil may achieve its control efficacy by interfering with olfactory signaling.

The conserved function of odorant receptor co-receptor (*Orco*) as a core olfactory co-receptor in insects has been extensively verified across diverse insects [17]. For example, Liu et al. reported that knockout of the *Orco* gene resulted in no response to rice volatile compounds and consequently no host plant preference in *Nilaparvata lugens* [18]. A previous study in *Spodoptera frugiperda* revealed that *Orco* knockout severely reduced the electrophysiological responses to plant volatiles and sex pheromones, and the mating behavior was fully disrupted in *Orco* knockout males [19], which was confirmed in another study [20]. The behavioral avoidance of *D. melanogaster* to plant-derived repellent compounds is also *Orco*-dependent, and repellency was reduced or abolished in two *Orco* mutants [21]. Furthermore, the loss of *Orco* function in *D. suzukii* prevents attraction to the odor of ripe strawberries, and the enhanced egg laying elicited by the odor was abolished [22]. These findings confirm that *Orco* serves as a core molecular element regulating its host selection, repellent responses and oviposition decisions. Given that *D. suzukii* relies on *Orco* for olfactory host location, and that many plant volatiles exert repellency through *Orco*-dependent pathways, sweet fennel essential oil may act as a repellent by interfering with this pathway. However, its bioactivity against *D. suzukii* and *Orco*-dependent mechanism remain elusive. Here, we assessed the repellency and oviposition deterrence of sweet fennel essential oil against *D. suzukii*, determined which constituents account for its repellent activity, and verified whether the repellency is mediated by *Orco*.

## 2. Materials and Methods

### 2.1. Insects

*D. suzukii*, kindly provided by Shaoying Wu (Hainan University), were collected from the field in Haikou, Hainan Province, China (20° N, 110° E) in 2018 and have been reared in laboratory conditions for more than five years. Both the wild-type and *Orco* knockout strains of *D. suzukii* were reared under identical laboratory conditions. Flies were maintained on standard cornmeal-yeast agar medium (Genesee Scientific, San Diego, CA, USA). Poplar wood wool (Hualeji, Hangzhou, China) and filter paper (Jiaojie, Dongguan, China) were added to rearing containers. All flies were kept in a growth chamber at 25 ± 0.5 °C under 65 ± 5% relative humidity with a 12 h light/12 h dark photoperiod.

### 2.2. Essential Oils and Chemicals

Sweet fennel oil was purchased from Dr Wong Co., Ltd. (Guangzhou, China). The primary constituent of sweet fennel essential oil, trans-anethole (purity ≥ 98%), 95% limonene and 97% fenchone, were obtained from Macklin Biochemical Co., Ltd. (Shanghai, China).

### 2.3. Dual-Choice Still-Air Assay

**Figure 1:**
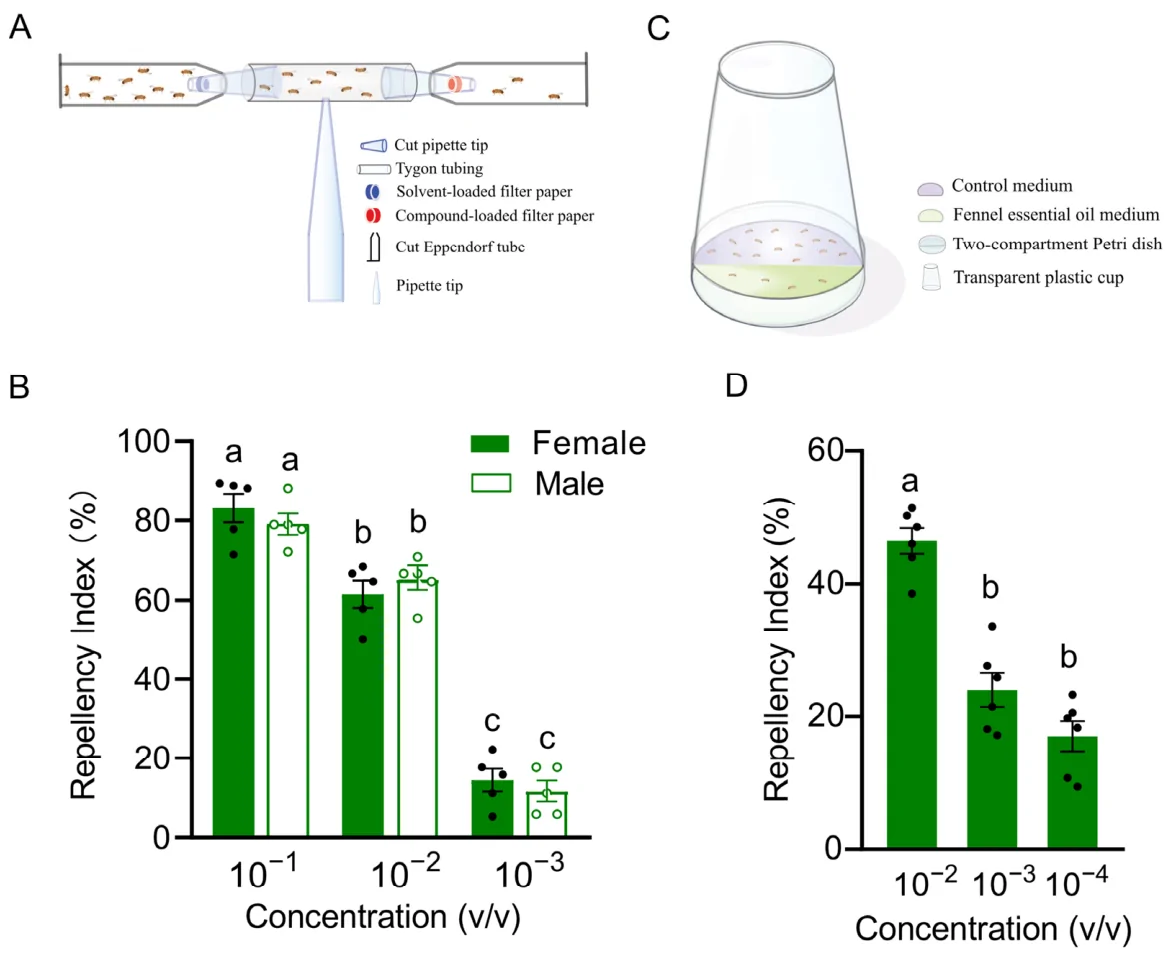
Repellency of sweet fennel essential oil against *D. suzukii*. (**A**) Schematic drawing of dual-choice still-air assay. (**B**) Repellency index of sweet fennel essential oil to female and male *D. suzukii* in dual-choice still-air assay (dilutions of sweet fennel essential oil were 10^−1^, 10^−2^ and 10^−3^ *v*/*v*, *n* = 5 for each dilution). (**C**) Schematic drawing of oviposition assay. (**D**) Repellency index of sweet fennel essential oil to *D. suzukii* in oviposition assay (dilutions of sweet fennel essential oil were 10^−1^, 10^−2^ and 10^−3^ *v*/*v*, *n* = 6 for each dilution). The number of eggs was as follows: 10^−2^ sweet fennel oil, 66.67 ± 2.89; control, 182.17 ± 3.32; 10^−3^ sweet fennel oil, 78.67 ± 3.88; control, 128.00 ± 3.03; 10^−4^ sweet fennel oil, 82.67 ± 2.06; control, 117.00 ± 4.86. Data are plotted as mean ± s.e.m. Dots denote the value of each repeat. Statistical analysis was conducted using one-way analysis of variance (ANOVA) followed by Tukey’s means separation test. Different letters indicate a significant difference between the repellency index of essential oils at different dilutions at *p* < 0.05.

The apparatus (Figure 1A) consisted of three arms. The central starting zone was constructed from a pipette tip, while the two test arms were assembled from 1.5 mL microcentrifuge tubes and Tygon tubing (inner diameter 6.35 mm, length 4 cm), connected via 1000 μL pipette tips and arranged in a perpendicular T-shape. Small holes were pierced in the lids of the microcentrifuge tubes for ventilation. Filter paper (3.2 cm × 0.8 cm) saturated with 50 μL of the test odorant (dissolved in paraffin oil) or solvent control was affixed to the inner walls of the microcentrifuge tubes. Twenty 3- to 5-day-old *D. suzukii* were carefully transferred into the apparatus through the Tygon tubing. All assays were conducted in an environmental chamber maintained at 25 ± 0.5 °C with 65 ± 5% relative humidity under a 12 h light/12 h dark photoperiod. The number of flies in each test arm was recorded after 24 h. The Repellency Index (RI) was calculated as (C − O)/(O + C) × 100, with O referring to the flies counted in the odorant-treated arm and C to those in the solvent-control arm. To eliminate potential side bias, the two arms of the T-maze apparatus were swapped during replications.

### 2.4. Two-Choice Oviposition Preference Assay

The oviposition assay was performed as described by Joseph et al. (2009), with minor modifications [23]. The two-choice oviposition preference apparatus (Figure 1C) consisted of a transparent hollow cylinder (height 80 mm, diameter 60 mm) with an agar plate placed at the bottom to serve as the oviposition substrate for *D. suzukii*. A total of 10 μL, 1 μL or 0.1 μL of essential oil was individually added to 100 mL of cooled molten fly food to obtain the 10^−2^, 10^−3^ or 10^−4^ treatments, respectively. Equal volumes (10 mL) of grape juice agar medium supplemented with sweet fennel essential oil and the corresponding control medium were poured into each side of a two-compartment plate to form the selective oviposition medium. Before initiation, adult flies were conditioned on an agar plate containing yeast paste for 48 h to enhance fecundity. Subsequently, 40 adult flies at a female-to-male ratio of 3:1 were introduced into the apparatus to select oviposition sites without artificial interference. The experimental conditions were consistent with those of the dual-choice still-air assay. The number of eggs laid on the agar plates in the treatment and control groups was counted after 24 h. The Repellency Index (RI) of the oviposition assay was calculated as in the dual-choice still-air assay.

### 2.5. Electroantennography (EAG)

The head of each 3- to 5-day-old *D. suzukii* adult was immobilized at the narrow end of a truncated 200 μL pipette tip, which was affixed to a microscope slide with dental wax. The antenna was extended stably on a micromanipulator stage via glass microelectrodes, which were filled with 0.5% polyvinylpyrrolidone (PVP) and 0.1% potassium chloride. The distal region of one antenna was connected to the recording electrode, while the reference electrode was inserted into the contralateral eye. The target compounds were serially diluted with paraffin oil. A total of 10 μL of each dilution was applied to a filter paper strip (0.2 × 30 mm), which was then loaded into a Pasteur pipette to assemble the odorant delivery system. The antenna was continuously flushed with a humidified airflow at a rate of 20 mL/s, and a 0.5 s pulse stimulus (flow rate: 2 mL/s) was triggered by a CS55 stimulus controller (Syntech, Kirchzarten, Germany). EAG signals were amplified and captured via an IDAC-4 interface (Syntech, Kirchzarten, Germany), and the data were analyzed using EAG-Pro version 2 (Syntech, Kirchzarten, Germany). Each compound was tested with only one stimulus administered per individual fly. EAG response values were calculated by subtracting the average response elicited by paraffin oil.

### 2.6. Orco Knockout Line (Orco^−/−^) Establishment

The *Orco* knockout line of *D. suzukii* was successfully established using the CRISPR–Cas9 gene editing system developed by Kistler et al. [24]. Specific sgRNAs targeting the first exon of the *Orco* gene (Gene ID: NM_001328601.1) were precisely designed using Zifit (https://chopchop.cbu.uib.no, accessed on 6 June 2026). The template of sgRNA was generated from a linear DNA template, which was amplified with primers (Table 1) by Phusion HighFidelity DNA Polymerase (NEB, Shanghai, China). The sgRNA was synthesized using the MEGAshortscript T7 High Yield Transcription Kit (Thermo Fisher Scientific, Shanghai, China) according to the manufacturer’s protocol. Cas9 protein was purchased from Invitrogen (Thermo Fisher Scientific, Shanghai, China). The injection was outsourced to a company (Fungene, Qidong, China). Progeny flies were genotyped using genomic PCR and sequencing. The primers used for genotyping are given in Table 1. Multigenerational crossing, screening and homozygous breeding were then performed to purify the genetic background. Four independent mutant individuals were used to generate the final homozygous knockout strain, *Orco*^−/−^.

### 2.7. Gas Chromatography–Mass Spectrometry (GC-MS)

Chemical constituents of sweet fennel essential oil were identified using a gas chromatography–mass spectrometry (GC-MS) system (QP2010 Plus, Kyoto, Japan) following the protocol described in our previous study [25]. An HP-5MS capillary column (30 m × 0.25 mm × 0.25 μm, Kyoto, Japan) was used for chromatographic separation. The temperature of the injector, transfer line and ion source were set to 250 °C, 290 °C and 230 °C, respectively. The column oven temperature was programmed: an initial temperature of 60 °C was held for 2 min, increased to 180 °C at 5 °C/min, then further raised to 280 °C at 10 °C/min. Helium served as the carrier gas with a constant flow rate of 1 mL/min. For sample pretreatment, essential oils were diluted to 1% (*v*/*v*) using ethyl acetate, and the injection volume was 1 μL. Mass spectrometry parameters were set as follows: scan rate of 0.5 scan/s, mass range of m/z 40–350, split ratio of 1:200, and ionization energy of 70 eV.

### 2.8. Statistical Analysis

GraphPad Prism 9.4.0 (GraphPad Software, San Diego, CA, USA) served as the tool for all statistical analyses, with the Shapiro–Wilk test adopted to assess the normality of the data. Repellency indices across diverse odorant dilutions were analyzed via one-way ANOVA, with Tukey’s multiple comparisons test or the Kruskal–Wallis test coupled with the Nemenyi test according to the parametric test prerequisites. Student’s unpaired *t*-test enabled direct comparison of EAG responses or repellency indices between the wild-type flies and *Orco*^−/−^ flies.

## 3. Results

### 3.1. Sweet Fennel Essential Oil Repels D. suzukii Adults

We conducted two behavioral assays, the dual-choice still-air assay and oviposition assay, to investigate the repellency of sweet fennel essential oil against *D. suzukii* adults. The results of the dual-choice still-air assay showed that sweet fennel essential oil exhibited a significant repellent effect against both male and female flies. It exhibited strong repellent effects at the 10^−1^ and 10^−2^ dilutions (*v*/*v*), while it had no repellency at the 10^−3^ (*v*/*v*) dilution (Figure 1B). Furthermore, a dose-dependent oviposition deterrent was also observed, with higher repellent activity at higher concentrations (Figure 1D).

### 3.2. Analysis of the Constituents of Sweet Fennel Essential Oil

Essential oil consists of multiple volatile constituents. In order to investigate the major constituents of sweet fennel oil, we analyzed its constituents using GC-MS. The most abundant constituent in sweet fennel essential oil was trans-anethole, at 87.67%. The constituents with the second and third highest contents were limonene and fenchone, with proportions of 2.56% and 1.82%, respectively (Table 2). The proportions of the other constituents were rather low.

### 3.3. Trans-Anethole Was Responsible for Repelling D. suzukii Adults

**Figure 2:**
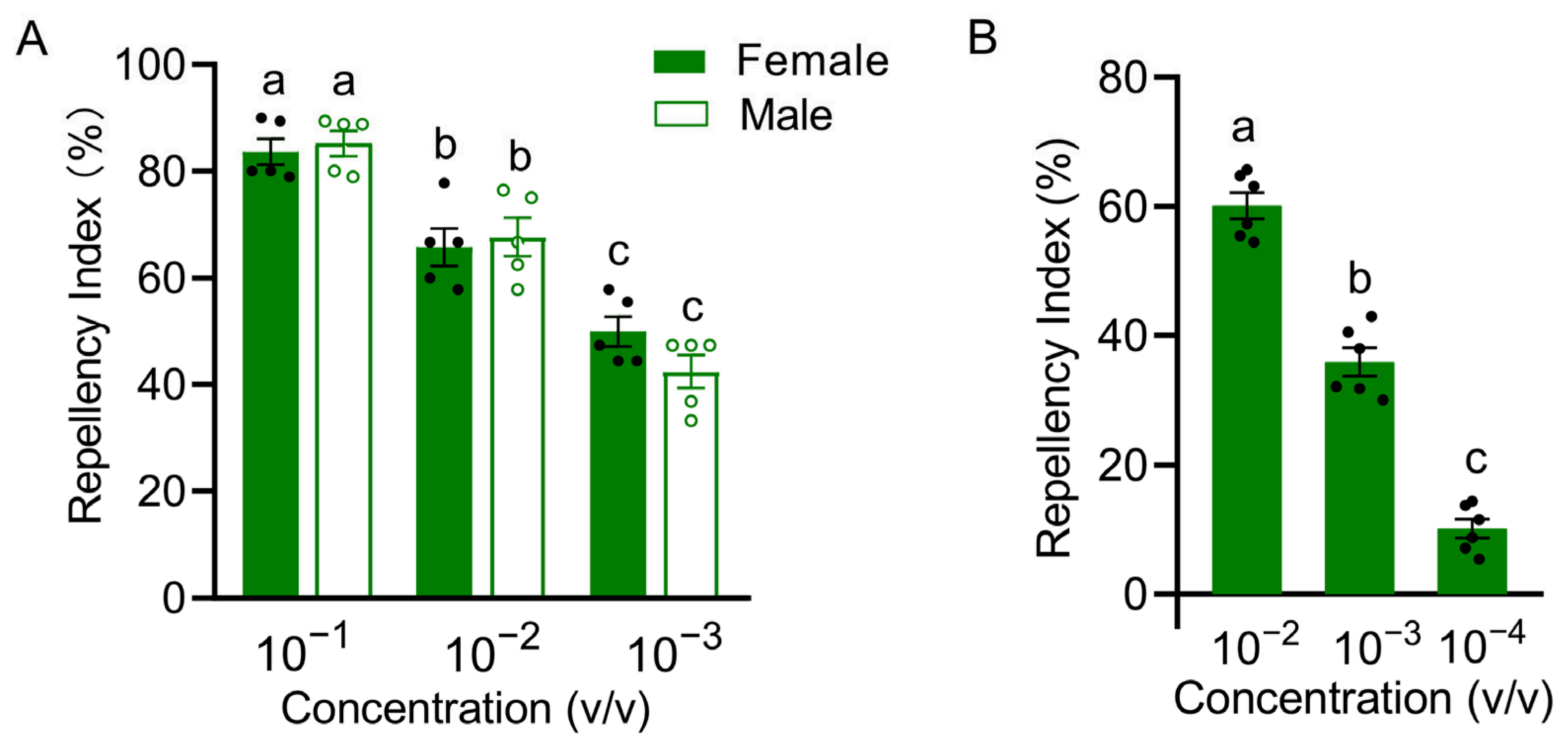
Repellency of trans-anethole against *D. suzukii*. (**A**) Repellency index of trans-anethole to female and male *D. suzukii* in dual-choice still-air assay (dilutions of trans-anethole were 10^−1^, 10^−2^ and 10^−3^ *v*/*v*, *n* = 6 for each dilution). (**B**) Repellency index of trans-anethole to *D. suzukii* in oviposition assay (dilutions of trans-anethole were 10^−2^, 10^−3^ and 10^−4^ *v*/*v*, *n* = 6 for each dilution). The number of eggs was as follows: 10^−2^ trans-anethole, 28.67 ± 2.88; control, 113.50 ± 5.26; 10^−3^ trans-anethole, 55.00 ± 2.37; control, 117.00 ± 5.81; 10^−4^ trans-anethole, 105.33 ± 4.23; control 129.00 ± 5.02. Data are plotted as mean ± s.e.m. Dots denote the value of each repeat. Statistical analysis was conducted using one-way analysis of variance (ANOVA) followed by Tukey’s means separation test. Different letters indicate a significant difference between the repellency index of essential oils at different dilutions at *p* < 0.05.

To investigate whether the major constituent, trans-anethole, was responsible for the repellency of sweet fennel oil, we evaluated the repellent activity of trans-anethole through the dual-choice still-air assay and oviposition assay. We found that trans-anethole exhibited significant repellency against flies in a dose-dependent manner in both assays. In the dual-choice still-air assay, trans-anethole showed repellency comparable to that of sweet fennel oil at 10^−1^ and 10^−2^ dilutions (*v*/*v*), and it maintained a repellency index of approximately 50% at the 10^−3^ dilution to both male and female flies (Figure 2A). Moreover, similar to that of sweet fennel essential oil, trans-anethole elicited significant oviposition deterrent at high concentrations (Figure 2B).

### 3.4. Knockout of Orco Abolished the EAG Responses of Sweet Fennel Essential Oil and Trans-Anethole

**Figure 3:**
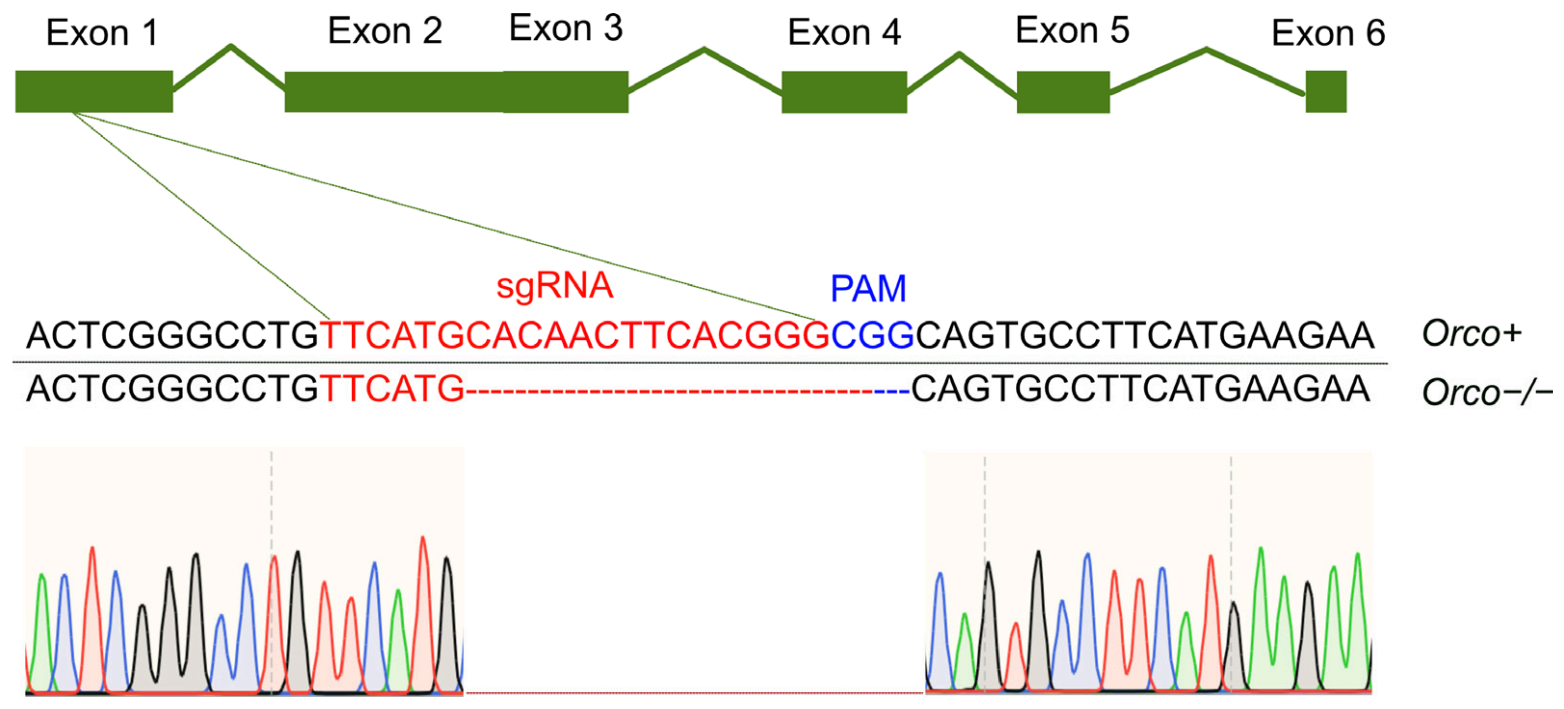
CRISPR–Cas9-based knockout of *Orco* gene. The sgRNA targeting *Orco* gene was designed on exon 1 of the *Orco* gene. Homozygous mutation of *Orco* with a 17 bp nucleotide deletion in the genome was confirmed via sequencing. The dotted portion in the *Orco*^−/−^ mutant indicates the nucleotide deletion.

In order to verify whether the repellency elicited by sweet fennel essential oil was mediated by the *Orco* pathway, we used CRISPR–Cas9 genome editing to generate an *Orco* functional null *D. suzukii* strain. Among the diverse mutations, one mutation with a 17 bp deletion in the first exon of *Orco*, which introduced a frameshift mutation and resulted in a loss-of-function protein, was used to generate the *Orco* knockout homozygous strain (*Orco*^−/−^) after four generations of hybridization (Figure 3).

**Figure 4:**
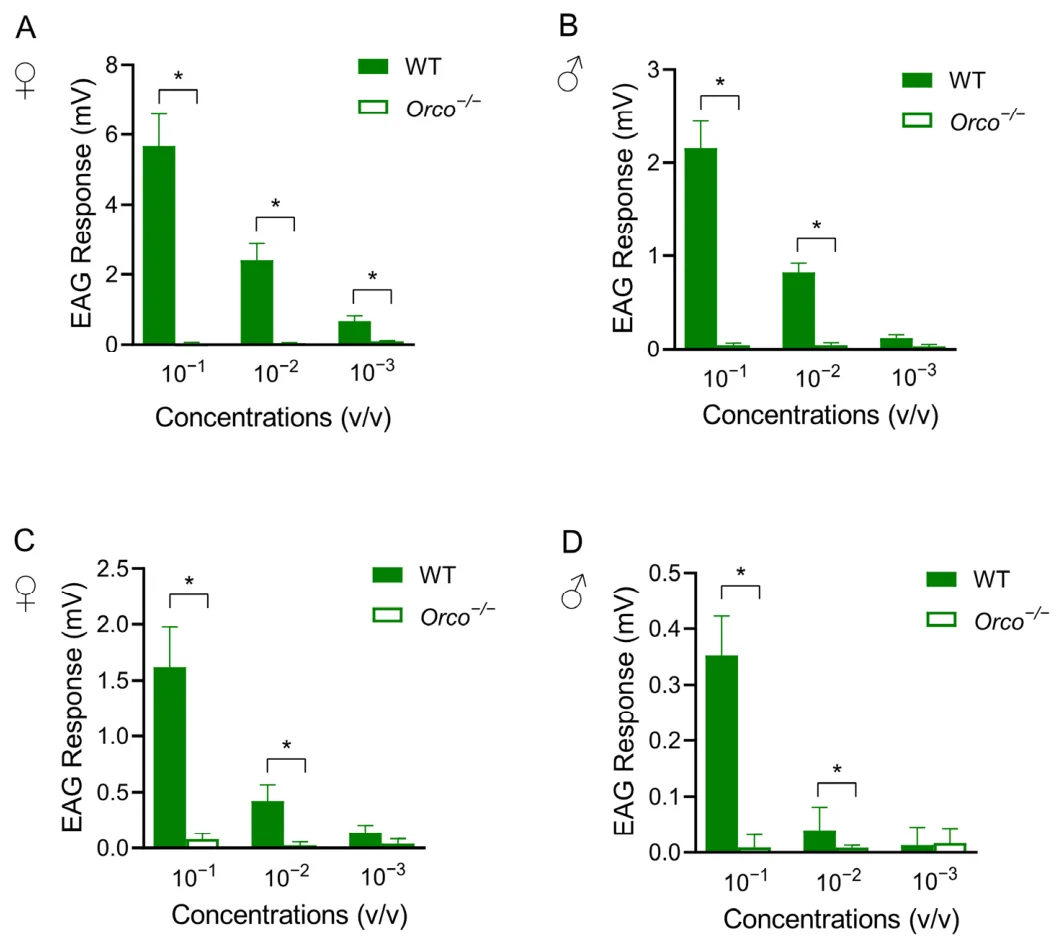
EAG responses of wild-type and *Orco*^−/−^ *D. suzukii* to sweet fennel essential oil and trans-anethole. (**A**,**B**) EAG responses of female and male *D. suzukii* to sweet fennel essential oil. (**C**,**D**) EAG responses of female and male *D. suzukii* to trans-anethole. Data are plotted as mean ± s.e.m., *n* = 8. Statistical analysis was conducted using Student’s unpaired *t*-test, and asterisks indicate a significant difference between the responses for wild-type and *Orco*^−/−^ flies at *p* < 0.05.

Then, we compared the EAG responses between wild-type and *Orco*^−/−^ flies to explore whether sweet fennel essential oil and trans-anethole elicited EAG responses in *D. suzukii* and whether the EAG responses were affected by *Orco* knockout. We found that both sweet fennel oil (Figure 4A,B) and trans-anethole (Figure 4C,D) were able to elicit dose-dependent EAG responses in wild-type *D. suzukii*. Additionally, the EAG responses from females were much larger than those from males. Futhermore, the EAG responses of sweet fennel essential oil were obviously larger than those of trans-anethole. However, the EAG responses disappeared in both female and male *Orco*^−/−^ flies at all concentrations, which was significantly different from those of wild-type *D. suzukii* (Figure 4).

### 3.5. Sweet Fennel Essential Oil and Trans-Anethole Failed to Elicit Repellency in Orco^−/−^ Flies

**Figure 5:**
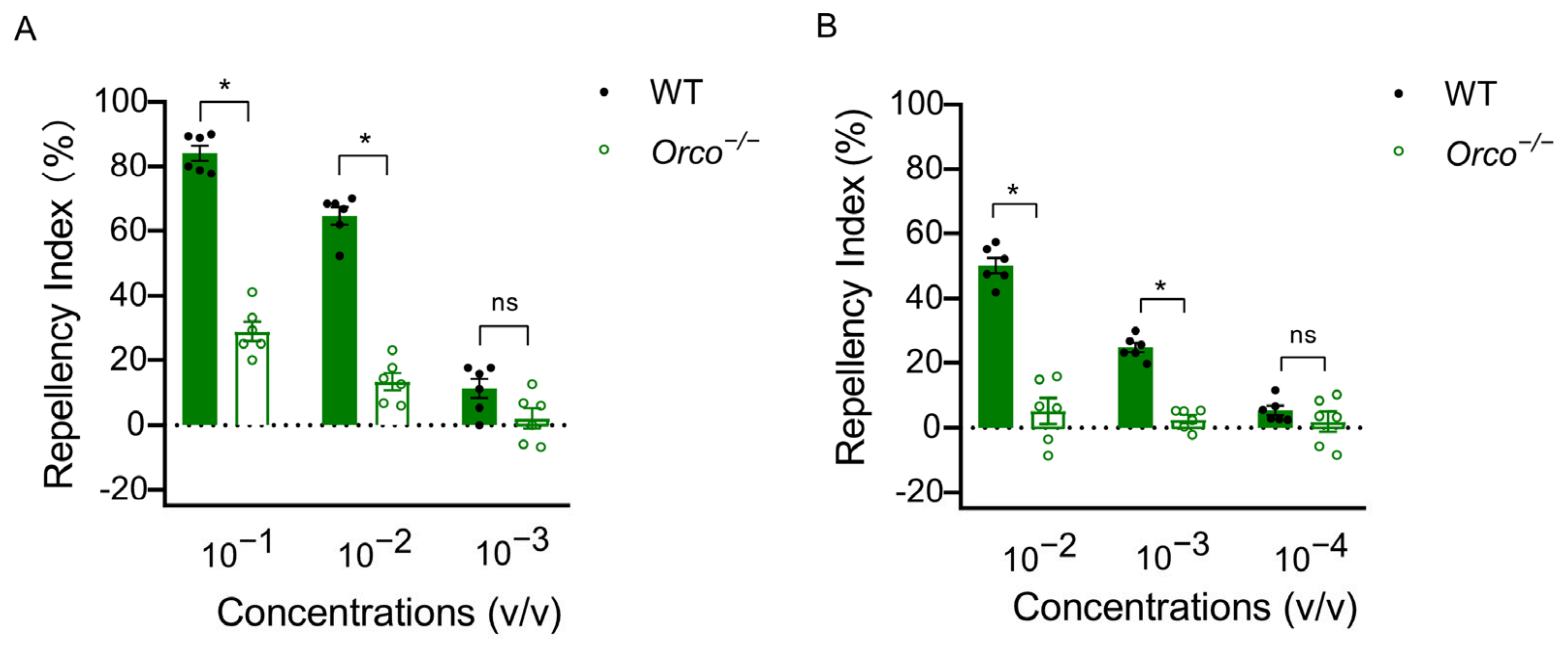
Repellency of sweet fennel essential oil against wild-type (WT) and *Orco*^−/^*^−^ D. suzukii*. (**A**) Repellency index of sweet fennel essential oil to *D. suzukii* in dual-choice still-air assay (dilutions of sweet fennel essential oil were 10^−1^, 10^−2^ and 10^−3^ *v*/*v*, *n* = 6 for each dilution). (**B**) Repellency index of sweet fennel essential oil to *D. suzukii* in oviposition assay (dilutions of sweet fennel essential oil were 10^−2^, 10^−3^ and 10^−4^ *v*/*v*, *n* = 6 for each dilution). The number of eggs was as follows: wild-type 10^−2^ sweet fennel oil, 56.00 ± 2.82; control, 169.00 ± 3.23; wild-type 10^−3^ sweet fennel oil, 89.33 ± 2.96; control, 147.83 ± 3.45; wild-type 10^−4^ sweet fennel oil, 105.83 ± 3.41; control, 117.50 ± 3.17; *Orco*^−/−^ 10^−2^ sweet fennel oil, 96.50 ± 3.11; control, 107.83 ± 6.78; *Orco*^−/−^ 10^−3^ sweet fennel oil, 108.00 ± 3.95; control, 114.17 ± 5.04; *Orco*^−/−^ 10^−4^ sweet fennel oil, 102.83 ± 3.22; control, 107.17 ± 5.27. Data are plotted as mean ± s.e.m. Dots denote the value of each repeat. Statistical analysis was conducted using Student’s unpaired *t*-test, and asterisks indicate a significant difference between the repellency in wildtype and *Orco*^−/−^ flies at *p* < 0.05.

**Figure 6:**
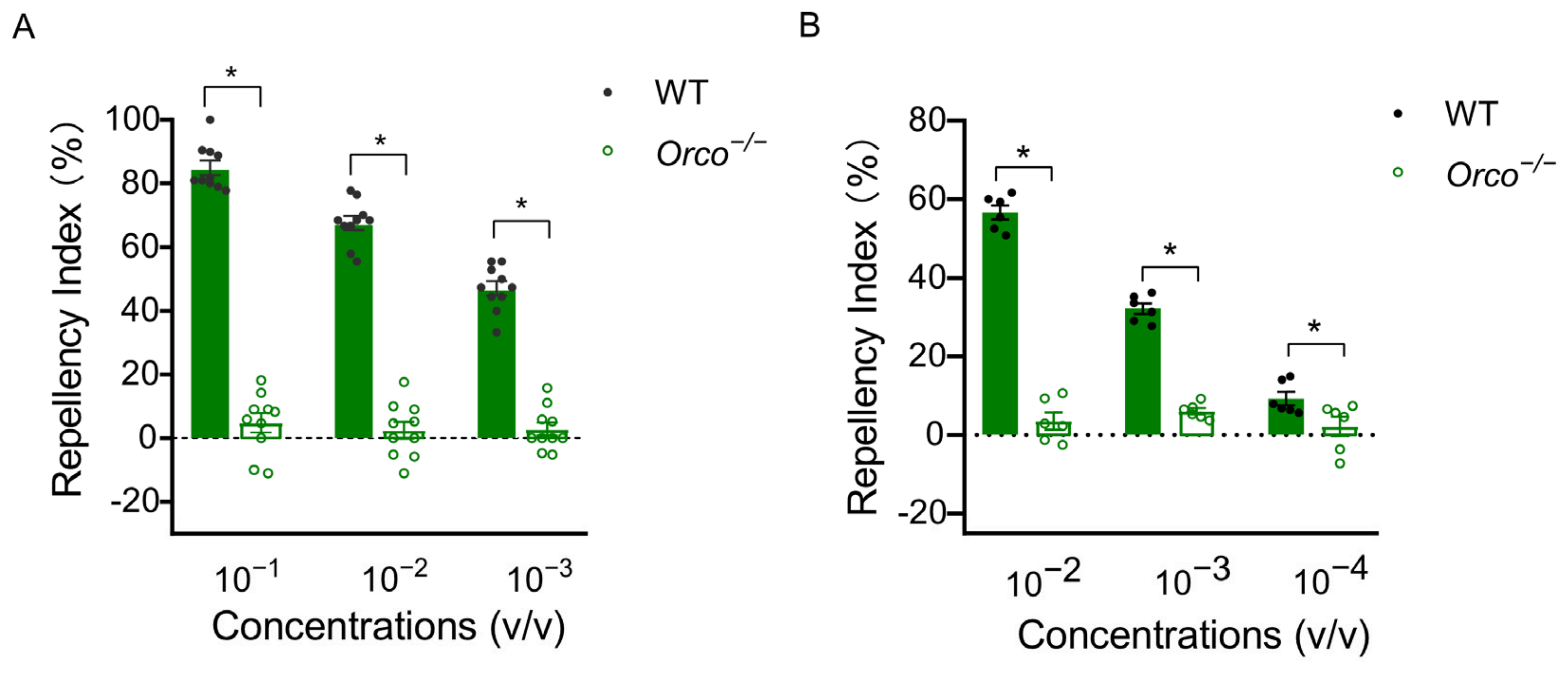
Repellency of trans-anethole against wild-type (WT) and *Orco*^−/−^ *D. suzukii*. (**A**) Repellency index of trans-anethole to *D. suzukii* in dual-choice still-air assay (dilutions of trans-anethole were 10^−1^, 10^−2^ and 10^−3^ *v*/*v*, *n* = 10 for each dilution). (**B**) Repellency index of trans-anethole to *D. suzukii* in oviposition assay (dilutions of trans-anethole were 10^−1^, 10^−2^ and 10^−3^ *v*/*v*, *n* = 6 for each dilution). The number of eggs was as follows: wild-type 10^−2^ trans-anethole, 37.67 ± 2.33; control, 135.33 ± 2.33; wild-type 10^−3^ trans-anethole, 74.50 ± 3.14; control, 145.00 ± 2.72; wild-type 10^−4^ trans-anethole, 100.50 ± 3.89; control, 121.17 ± 4.82; *Orco*^−/−^ 10^−2^ trans-anethole, 37.83 ± 1.54; control, 40.83 ± 6.24; *Orco*^−/−^ 10^−3^ trans-anethole, 99.50 ± 3.43; control, 112.67 ± 5.15; *Orco*^−/−^ 10^−4^ trans-anethole, 93.50 ± 4.35; control, 97.33 ± 1.78. Data are plotted as mean ± s.e.m. Dots denote the value of each repeat. Statistical analysis was conducted using Student’s unpaired *t*-test, and asterisks indicate a significant difference between the repellency in wild-type and *Orco*^−/−^ flies at *p* < 0.05.

**Figure 7:**
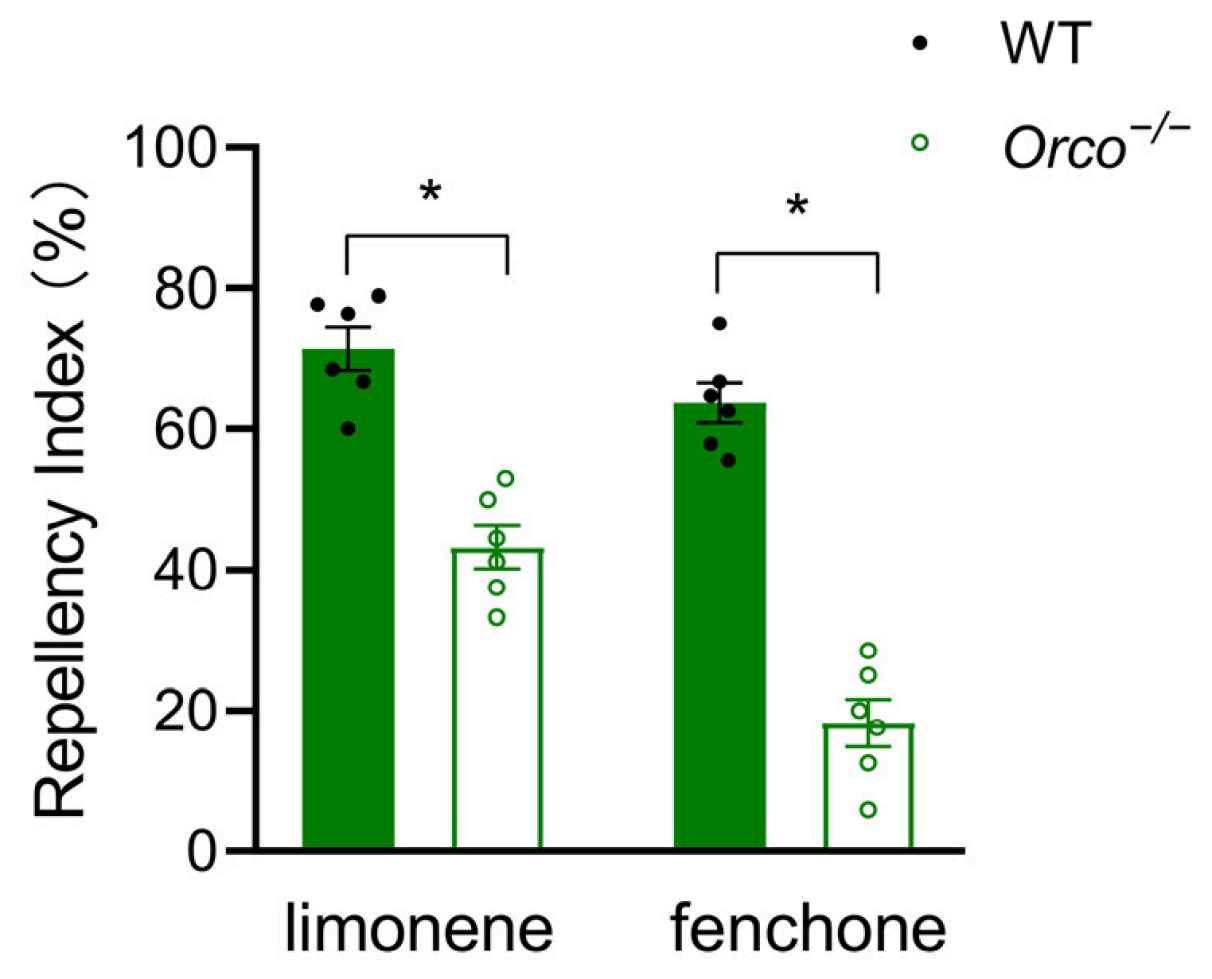
Repellency of limonene and fenchone against wild-type (WT) and *Orco*^−/−^ *D. suzukii* in dual-choice still-air assay. The dilution was 10^−1^ *v*/*v*. Data are plotted as mean ± s.e.m. Dots denote the value of each repeat. Statistical analysis was conducted using Student’s unpaired *t*-test, and asterisks indicate a significant difference between the repellency in wild-type and *Orco*^−/−^ flies at *p* < 0.05.

Behavioral assays were subsequently conducted with *Orco*^−/−^ flies to determine the role of *Orco*. Since the EAG responses were abolished in both male and female *Orco*^−/−^ files, we conducted the dual-choice still-air assay with mixed sexes in a 1:1 ratio. Inconsistent with those of the wild-type strain, the *Orco*^−/−^ flies showed no significant avoidance to sweet fennel essential oil in the dual-choice still-air assay, except for 10^−1^ dilution (*v*/*v*), which exhibited a low Repellency Index of approximately 25% (Figure 5A). In addition, no significant oviposition deterrence was observed at any of the dilutions (Figure 5B). Moreover, we examined the repellency of trans-anethole against the *Orco*^−/−^ strain. We found that the repellency was abolished in both assays at all dilutions (Figure 6), which was significantly different from that of wild-type flies. Finally, we also tested the repellency of minor constitutes, limonene and fenchone, against *D. suzukii* in the dual-choice still-air assay. Contrary to trans-anethole, both compounds exhibited low repellency to flies (Figure 7).

## 4. Discussion

Plant essential oils have become promising and eco-friendly alternatives to conventional chemical pesticides owing to their environmental compatibility and low toxicity to mammals. Numerous essential oils have demonstrated strong repellency against *D. suzukii*, such as neem oil [26], *Origanum vulgare* oil, *Artemisia herba-alba* oil [27], *Cinnamomum verum* oil and *Cymbopogon citratus* oil [28], *Litsea cubeba* essential oil and *Pectis brevipedunculata* essential oil [29,30]. Fennel essential oil exhibited excellent repellent effects against *Musca domestica* [31], *Sitophilus zeamais*, *Cryptolestes ferrugineus* [32], *Rhyzopertha dominica*, and *Tribolium confusum* [33]. In the present study, we found that sweet fennel essential oil exerted significant repellent and oviposition-deterrent effects on adult *D. suzukii*, indicating the promising potential of fennel oil for pest management.

GC-MS analysis demonstrated that our tested sweet fennel essential oil belonged to the chemotype with high trans-anethole content [34,35,36]. As a typical monoterpenoid compound, trans-anethole has been proven to repel several agricultural pests and sanitary pests, including *Plodia interpunctella* [37], *T. castaneum* [38], *M. domestica* [39] and *Periplaneta americana* [40]. Existing evidence shows that star anise essential oil, which is rich in trans-anethole, displays strong repellency against *D. melanogaster* larvae [41]. Our results indicated that trans-anethole also exerts prominent repellent effects on adult *D. suzukii*, suggesting that essential oils abundant with trans-anethole might be excellent repellents against *Drosophila* species. In addition, pure trans-anethole exhibited comparable repellent activity to that of sweet fennel essential oil, further confirming that trans-anethole is the core active component responsible for the repellent and oviposition-deterrent effects of sweet fennel essential oil. Consistent with our previous study, the results revealed that EAG responses of females to odorants were stronger than those of males [42]. Here, we also compared the repellent efficiency of essential oil between the sexes, but no differences were found.

Feeding, mating, oviposition and other vital behaviors in insects are highly dependent on their olfactory systems. Our results showed that both sweet fennel essential oil and trans-anethole elicited obvious EAG responses in *D. suzukii*, indicating that both substances can be recognized by olfactory sensilla on fly antennae. Meanwhile, the EAG amplitude induced by sweet fennel oil was higher than that induced by constituent trans-anethole, which is consistent with our previous study in which clary sage oil elicited a larger EAG response than constituents linalyl acetate and linalool [25]. These results suggest that the olfactory system of *D. suzukii* is more sensitive to complex mixtures of essential oil components, and other minor volatile components might be also involved in the olfactory behavior.

A previous study indicated that *D. melanogaster* can suppress oviposition attraction to volatiles of ripe strawberries via an *Orco*-dependent pathway. However, the egg-laying behavior of *D. suzukii* was not affected by loss of *Orco* function; the *Orco* knockout strain remained attracted to both ripe strawberry odor substrates and the synthetic two-component blend [43]. These findings are discrepant with another study, which revealed that the loss of *Orco* function in *D. suzukii* prevented attraction to the odors of ripe strawberries, while besides *Orco*-mediated olfaction, contact chemosensation was also involved in the oviposition behavior of *D. suzukii* on ripe fruit [22]. *Orco* knockout eliminated the chemotactic response of *D. suzukii* to phenylacetaldehyde, a characteristic host volatile, and reduced olfactory sensitivity to acetic acid, a fermentation marker, thereby blocking the precise localization of ripe fruits at the peripheral sensory level [44]. Similar with that reported in *D. melanogaster*, *Orco* mediated the repellency of natural insecticide pyrethrum [45] and odorants including menthone, (R)-(+)-limoneneor and (+)-fenchone [21]. In this study, we also observed that the attraction of apple cider vinegar (ACV) disappeared in *Orco*^−/−^ flies (Figure S1). Moreover, we found that the egg-laying ability of flies was not affected by *Orco* knockout, except at a high dose of trans-anethole, which slightly reduced egg numbers. Overall, our results showed that *Orco* knockout dramatically reduced the EAG responses and avoidance behaviors of flies to both sweet fennel essential oil and trans-anethole. These findings confirmed that the olfactory co-receptor *Orco* serves as a core target mediating odor recognition and the subsequent repellent behaviors.

Besides *Orco*-dependent signaling, other pathways might be involved in odorant recognition and behaviors. Flies may adopt distinct neural regulatory patterns in response to complex essential oils versus single chemical monomers. For instance, the dopamine signaling pathway specifically participates in regulating fly chemotaxis toward complex odors from a larval food or yeast paste, but it has no significant effect on responses to individual compounds [41]. Transient receptor potential ankyrin 1 (TRPA1) channels are nociceptors that can be activated by noxious heat or chemicals, and TRPA1 channels typically trigger repellency in mosquitoes [46,47,48], as well as in *T. castaneum* [49] and *Lygus hesperus* [50]. In this study, we found that the repellency of sweet fennel oil at high concentrations was not completely abolished in the T-maze assay, as well as those in minor constitutes, limonene and fenchone, indicating that *Orco*-independent mechanisms might also contribute. The complex oil likely activates a broad array of olfactory receptors or other pathways such as dopamine transmission and TRPA1 channels to trigger avoidance behavior in insects.

## 5. Conclusions

In conclusion, sweet fennel essential oil and the bioactive constituent trans-anethole evoked strong repellency in *D. suzukii*. Upon loss of *Orco* function, the EAG responses and repellency were significantly reduced, indicating that *Orco* is involved in the avoidance behavior of *D. suzukii*. Our results provide evidence that sweet fennel essential oil and trans-anethole are promising candidates for development as effective fly repellents and lay the foundation for exploring the molecular olfactory mechanism of natural plant essential oil in insects.

## Figures and Tables

**Table 1:** The primers used in this study.

Primer Name	Sequence (5′-3′)
For sgRNA synthesis	
sg*Orco*-F	TAATACGACTCACTATATTTATGCACAACTTCACGGGGTTTTAGAGCTAGAAATAGCAAGTTAAAATAA
sg*Orco*-R	AGCACCGACTCGGTGCCACTTTTTCAAGTTGATAACGGACTAGCCTTATTTTAACTTGCTATTTCTAGCTCTAAAAC
For genotyping	
*Orco*-F	TAAACGGACACTAACGATTC
*Orco*-R	GGATACATATTGTGCCAGTT

**Table 2:** Constituents with relative abundance > 0.5% in sweet fennel essential oil.

No	RI	RI Lit	Compound	CAS	Relative Percentage (%)
1	1039	1031	Limonene	138-86-3	2.56
2	1092	1096	Fenchone	1195-79-5	1.82
3	1295	1301	Trans-anethole	4180-23-8	87.67

Note: Retention index (RI) was determined relative to the retention times of a homologous series of n-alkanes C7–C30 on an HP-5MS column. RI Lit values were obtained from the NIST Chemistry WebBook. Relative proportions of the essential oil constituents are expressed as percentages.

## Data Availability

The data presented in this study are available upon request from the corresponding author.

## Supplementary Materials

The following supporting information can be downloaded at: https://www.mdpi.com/article/10.3390/insects17090957/s1, Figure S1: Preference index of apple cider vinegar against *D. suzukii*.
