# Supplementary material for: Sweet Fennel Essential Oil Repels Drosophila suzukii via an Orco-Mediated Pathway
Source: Insects. 2026 Sep 14;17(9):957. doi: 10.3390/insects17090957 (PMC13607007; doi:10.3390/insects17090957)
Supplement: Supplementary file 1 [file insects-17-00957-s001.zip › insects-4426827-supplementary.pdf]

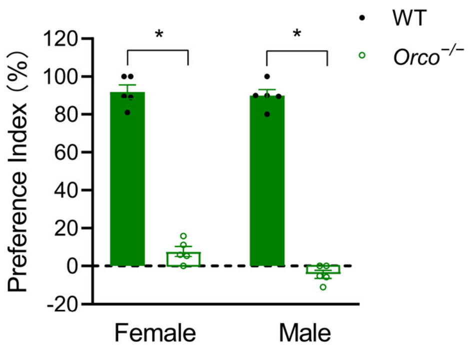

Figure S1. Preference index of apple cider vinegar against wild-type (WT) and *Orco*$^{-/-}$ *D. suzukii*. The concentration of apple cider vinegar was 10^{-1} v/v. n = 5, data are plotted as mean ± s.e.m. Dots denote the value of each repeat. Statistical analysis was conducted by Student's unpaired t-test, and asterisks indicate a significant difference between the response for wild-type and *Orco*$^{-/-}$ flies at $p < 0.05$.
